# Cognition, Reserve, and Amyloid Deposition in Normal Aging

**DOI:** 10.1002/ana.21904

**Published:** 2009-10-27

**Authors:** Dorene M Rentz, Joseph J Locascio, John A Becker, Erin K Moran, Elisha Eng, Randy L Buckner, Reisa A Sperling, Keith A Johnson

**Affiliations:** 1 Department of Neurology, Brigham and Women's Hospital, Harvard Medical SchoolBoston, MA; 2 Department of Neurology, Massachusetts General Hospital, Harvard Medical SchoolBoston, MA; 3 Department of Brain and Cognitive Sciences, Massachusetts Institute of TechnologyCambridge, MA; 4 Department of Radiology, Massachusetts General Hospital, Harvard Medical SchoolBoston, MA; 5 Department of Psychology and Center for Brain Science, Harvard UniversityCambridge, MA; 6 Department of Psychiatry, Massachusetts General Hospital, Harvard Medical SchoolBoston, MA; 7 Athinoula A. Martinos Center for Biomedical ImagingCharlestown, MA; 8 Howard Hughes Medical InstituteCambridge, MA

## Abstract

**Objective:**

To determine whether amyloid deposition is associated with impaired neuropsychological (NP) performance and whether cognitive reserve (CR) modifies this association.

**Methods:**

In 66 normal elderly controls and 17 patients with Alzheimer disease (AD), we related brain retention of Pittsburgh Compound B (PiB) to NP performance and evaluated the impact of CR using education and American National Adult Reading Test intelligence quotient as proposed proxies.

**Results:**

We found in the combined sample of subjects that PiB retention in the precuneus was inversely related to NP performance, especially in tests of memory function, but also in tests of working memory, semantic processing, language, and visuospatial perception. CR significantly modified the relationship, such that at progressively higher levels of CR, increased amyloid deposition was less or not at all associated with poorer neuropsychological performance. In a subsample of normal controls, both the main effect of amyloid deposition of worse memory performance and the interaction with CR were replicated using a particularly challenging memory test.

**Interpretation:**

Amyloid deposition is associated with lower cognitive performance both in AD patients and in the normal elderly, but the association is modified by CR, suggesting that CR may be protective against amyloid-related cognitive impairment. ANN NEUROL 2010;67:353–364

Amyloid-beta deposition is a central pathophysiological marker of Alzheimer disease (AD) but, at autopsy, a correlation of dementia severity with amyloid burden has generally not been found, raising questions about its relationship to disease progression.^1^–^6^ With the advent of in vivo amyloid imaging using positron emission tomography (PET) with Pittsburgh Compound B (PiB), amyloid deposition has been reported in a variety of clinical conditions and in subjects with a wide range of clinical impairments. Specific binding and retention of PiB in cortex has now been demonstrated in patients with AD,^7^–^11^ dementia with Lewy bodies,^11^–^13^ cerebral amyloid angiopathy,^9^, ^14^ and subsets of mildly impaired^15^, ^16^ or apparently unimpaired normal control subjects (NCS).^9^, ^16^–^20^ The presence of amyloid-beta deposition in clinically normal individuals suggests that PiB retention might be an antecedent marker along the AD trajectory.^15^, ^16^ Normal individuals with elevated PiB retention showed decreased brain volumes, reduced cortical thickness, and abnormal functional activity during memory encoding relative to peers.^21^–^23^ However, further research is required to clarify the relation of PiB retention to clinical impairment and to elucidate the influence of mediating factors. In particular, normal individuals with PiB deposition may show subtle signs of cognitive change if amyloid-beta deposition marks an early stage of pathophysiological change.

One of these potentially mediating factors is cognitive reserve (CR), which we refer to as a broad concept conferring a reduced susceptibility to impairment due to individual characteristics such as increased synaptic or neuronal capacity,^24^, ^25^ greater efficiency engaging brain networks, or the use of alternative strategies.^26^–^28^ We hypothesize that these mechanisms mediating why some individuals better tolerate disease burden likely arise from multiple factors. Originally proposed to account for the finding of substantial AD pathology at postmortem in individuals considered normal during life,^24^, ^29^–^31^ the concept of CR was broadened by Stern and colleagues, who reported that higher levels of premorbid education and occupational attainment in AD patient groups matched for overall dementia severity were related to lower levels of temporoparietal metabolism.^32^–^35^ These reports support the notion that individuals with greater CR exhibit reduced susceptibility to dementia, possibly due to tolerance of underlying AD pathology. More recently, the impact of CR has been reported in studies of PiB PET amyloid burden in AD patients using educational attainment as the proxy of CR.^36^, ^37^

Since amyloid-beta pathology develops several years before cognitive impairment, diagnostic tools for prodromal AD have been actively sought, and amyloid imaging has emerged as a potentially useful biomarker. To fully understand the association of amyloid with impairment, the mediating effect of reserve should be evaluated formally, including in individuals who are clinically normal. To accomplish this, we evaluated PiB retention as a continuous variable across wide ranges of amyloid burden and cognitive function, including normal subjects and patients with mild AD, and explored both education and an estimate of ability level (American National Adult Reading Test [AMNART] intelligence quotient [IQ]) as proxies of CR.

## Subjects and Methods

### Subjects

Eighty-three subjects enrolled in longitudinal studies of aging and dementia at the Massachusetts General Hospital and Brigham and Women's Hospital were studied using protocols and informed consent procedures approved by the Partners Human Research Committee. Sixty-six were NCS (Clinical Dementia Rating [CDR] = 0)^38^ and had a mean (standard deviation [SD]) age of 73.9 (8.1) years with a range from 46.2 to 92.4 years. We had a comparison group of 17 patients who met National Institute of Neurological and Communicative Disorders and Stroke criteria for AD (CDR ≥ 1)^39^ and had a mean age of 66.5 (11.5) years with a range from 49.9 to 85.6 years (Table 1). A review of history and functional performance as well as physical and neurological examinations confirmed each diagnosis or status. None of the participants had a history of alcoholism, drug abuse, head trauma, or current serious medical or psychiatric illness.

**Table 1:** Demographic Statistics and Neuropsychological Performance

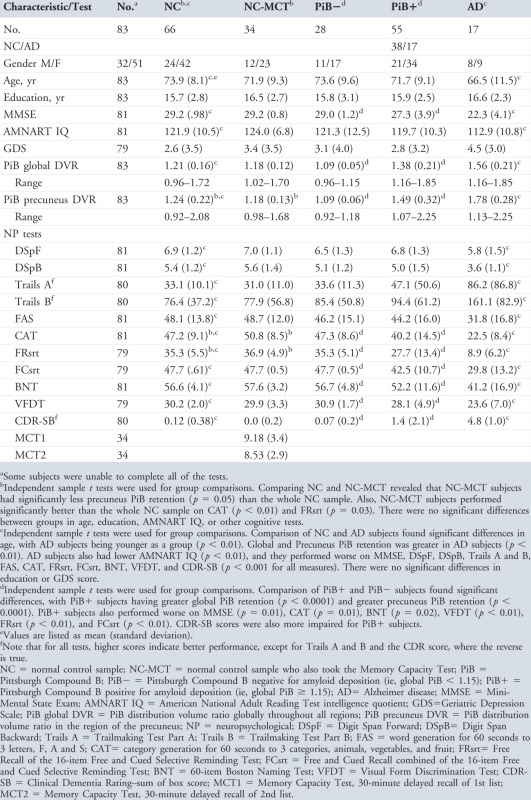

### Neuropsychological Evaluation

Subjects underwent functional assessments including subject and informant ratings on the CDR^38^ and the Geriatric Depression Scale.^40^ A core set of 11 cognitive assessments included the Mini-Mental State Exam (MMSE),^41^ Digit Span Forward and Backward,^42^ Trails A and B,^43^ Controlled Oral Word Fluency to the letters F-A-S (FAS),^44^ Category Generation to animals, vegetables, and fruit (CAT),^45^ the 16-item, 3-trial version of the Free and Cued Selective Reminding Test (FCSRT)^46^ utilizing Free Recall (FRsrt) and Cued Recall (FCsrt) subtests, 60-item Boston Naming Test (BNT),^47^ and the Visual Form Discrimination Test (VFDT).^44^ An additional, more challenging neuropsychological (NP) test, the Memory Capacity Test (Buschke 2005, personal communication) was administered to a consecutively chosen subset of 34 NCS. This test was chosen because it does not have the same ceiling effect as the FCSRT in normal controls. We evaluated first and second list learning during free recall at 30 minutes (Memory Capacity Test [MCT]). Group mean test scores are given in Table 1. NP test intercorrelations were *r* = 0.4 to 0.75. The mean (SD) time between PET imaging and testing was 0.90 (1.9) months (except for administration of the MCT, which was 8.03 [9.1] months).

### Cognitive Reserve

To estimate CR, we employed 2 variables, years of education and ability level as determined by AMNART,^48^ a pronunciation list of 50 irregular words that is highly correlated with measured intelligence on the Wechsler Adult Intelligence Scale-revised verbal IQ (*r* = 0.80–0.95).^48^–^51^ However, in a preliminary analysis of education as a CR variable, an independent samples *t* test in the entire sample (N = 83) revealed a significant gender bias on education with women (mean = 15.0, SD = 2.6) being less educated than men (mean = 17.2, SD = 2.3; *p* < 0.001, 2-tailed) that also occurred in the normal control sample (women: mean = 14.9, SD = 2.7; men: mean = 17.0, SD = 2.5; *p* < 0.005, 2-tailed). There was no significant gender bias for AMNART IQ in either the entire sample (N = 83; women: mean = 119.5, SD = 11.9; men: mean = 121.4, SD = 9.7; *p* = 0.44, 2-tailed) or in the normal control sample (women: mean = 121.1, SD = 11.1; men: mean = 123.4, SD = 9.4; *p* = 0.40, 2-tailed). We tested our main hypotheses with both education and AMNART IQ and elected not to covary gender, because it would have statistically oversaturated our models.

One potential problem with using AMNART as a proxy of CR is that predictions may underestimate premorbid IQ in patients with AD.^52^–^54^ To resolve this, we linearly regressed AMNART on MMSE as a measure of dementia severity^52^ (*r* = 0.42, *p* < 0.001) and used the residual (rAMNART) as the IQ score to estimate CR. For NCS this adjustment was not necessary, as the mean (SD) MMSE was 29.2 (0.98), and AMNART and rAMNART correlated at *r* = 0.99, but for simplicity, we report rAMNART for all analyses, including those involving only normal control subjects.

### PET Imaging

PiB was prepared as described by Mathis et al,^55^ and PiB PET acquisitions were performed as described previously.^9^, ^13^ Following a transmission scan, 8.5–15mCi 11C-PiB was injected as a bolus and followed immediately by a 60-minute dynamic acquisition. PiB PET data were reconstructed with ordered set expectation maximization, corrected for attenuation. Each frame was evaluated to verify adequate count statistics and absence of head motion. The Logan graphical analysis method^56^, ^57^ with cerebellar cortex as the reference tissue input function was used to evaluate specific PiB retention expressed as the distribution volume ratio (DVR).^9^, ^13^, ^19^, ^58^–^61^

### Image Analysis

We calculated the DVR (with cerebellar gray reference) in aggregate cortical regions of interest (ROIs), global mean cortical, bilateral precuneus, lateral frontal (superior and middle frontal gyri), and occipital (superior, middle, and inferior occipital gyri),^62^ as described previously.^9^, ^13^ The precuneus, including the posterior cingulate, was selected because of its prominent, early amyloid deposition,^19^ and because of its role in memory function^17^; lateral frontal was chosen because it has been consistently reported to contain very high levels of PiB retention^13^, ^19^, ^60^; occipital was chosen as a control region to represent intermediate levels of PiB retention.^13^

### Data Analysis

We used canonical correlation analysis, a multivariate technique, to specifically search for the linear combination (“canonical variate”) of the 11 NP tests that related most strongly to a corresponding linear combination of the set of predictors under consideration.^63^ The canonical correlation provided a single, unbiased test of statistical significance, which takes into account the sample size, the number of variables in the analysis, and intercorrelations between and within sets of them, as well as the fact that it is a pair of linear combinations prederived to maximally correlate whose correlation is being tested for significance. Note that although >1 canonical correlation can be sequentially derived, uncorrelated with those previously derived, each canonical variate is initially evaluated to assess whether the magnitudes and signs of its loadings (ie, the correlation of each constituent variable with the canonical variate) suggest a reasonable and substantive meaning (see Supplemental Table S1 for a more detailed explanation).^63^ Here the putative set of predictors were: age, years of education, rAMNART, PiB retention, and the interaction (cross-product) of rAMNART with PiB retention. The interaction is sensitive to any differential relations of PiB retention to the NP tests dependent on level (strata) of CR. We further characterized any discovered significant canonical relation with post hoc multiple regressions relating (1) the canonical variate of the 11 NP tests to the set of predictors and (2) each individual NP test to the same set of predictors. We performed this same set of analyses in the full sample (n = 83) and in 2 subsets of subjects, (1) those with high amyloid burden (PiB-positive, defined as being within the range of AD, ie, global mean PiB ≥ 1.15, n = 51; CDR0 n = 36; CDR1 n = 15) and (2) those in the subset of NCS (n = 66). Finally, for the MCT, which was administered to a subset (n = 34) of the NCS, we performed regression analyses with the same set of putative predictors as above: age, years of education, rAMNART, PiB retention, and the interaction (cross-product) of rAMNART with PiB retention. (These 34 subjects were similar in age, gender, education, AMNART IQ, MMSE, and precuneus PiB DVR to the larger NCS sample, n = 66; see Table 1.) The analyses were performed with SPSS v17.0 (SPSS Inc., Chicago, IL) and SAS v9.1.3 (SAS Institute Inc., Cary, NC).

## Results

### Association of Precuneus Amyloid Deposition and NP Performance as Modified by CR across All Subjects (n=83)

We initially explored whether precuneus PiB retention was associated with impaired NP performance using a canonical correlation analysis across both normal subjects and patients with AD. The canonical correlation analysis of the set of 11 NP tests versus the set of predictors revealed 2 canonical correlations that were individually significant (*p* < 0.001) and of about equal strength (see Supplemental Table S1). (Because some variables were non-normally distributed in violation of test assumptions, the statistical significance of all relevant canonical correlations in this study were confirmed with nonparametric permutation tests of 1,000 resamples, all of which returned *p* < 0.003.) The first pair of canonical variates had weaker loadings on the NP tests, whereas the second pair of canonical variates (canonical *r* = 0.70) were loaded moderately to highly on all of the NP tests (loadings for each NP test are given in Supplemental Table 2). The corresponding variate for the predictor set showed a strong negative loading for precuneus PiB retention that is consistent with increasing amyloid burden and worsening NP performance. However, there was also a strong positive loading for the interaction of PiB with rAMNART consistent with a moderating effect of CR, whereby the negative relation of amyloid to NP performance became less and less negative at progressively higher strata of CR.

**FIGURE 1:**
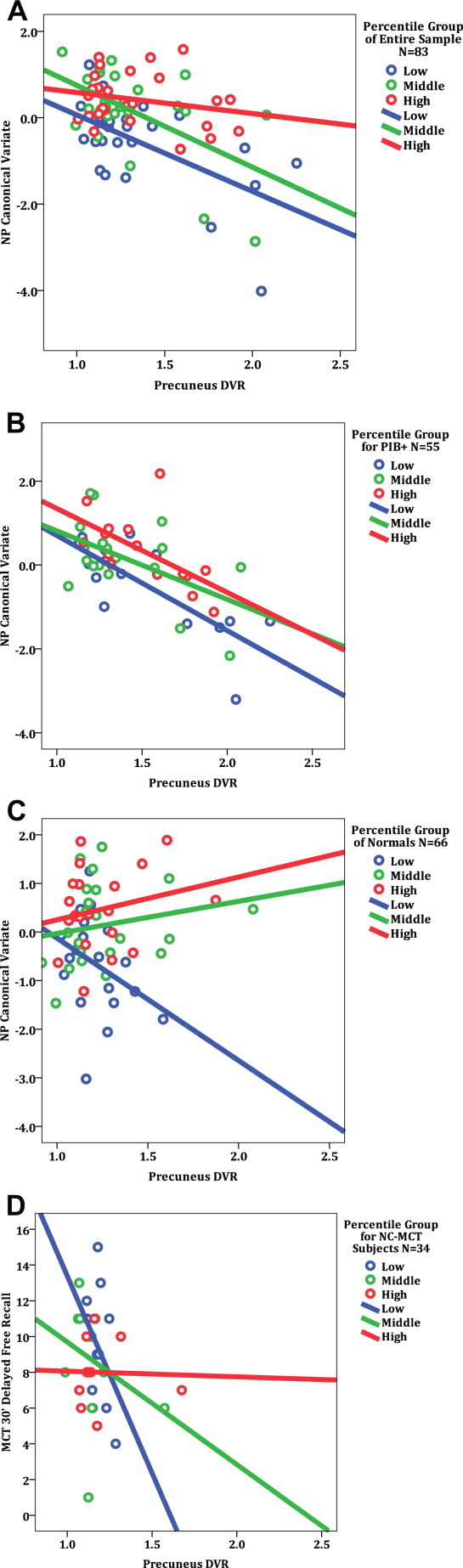
Neuropsychological performance versus precuneus Pittsburgh Compound B (PiB) retention. In each panel, subjects are color-coded by tertile strata of increasing cognitive reserve (CR), expressed as residual American National Adult Reading Test (AMNART) intelligence quotient (IQ): low, medium, and high. Solid lines represent the ordinary least squares regression lines for each stratum. Note that data points shown are not adjusted for age or education, as in the regression analyses (see Table 2). (A) In the full sample of subjects (N = 83), neuropsychological (NP) performance represented by the canonical variate was inversely related to PiB retention, but the effect was attenuated at higher levels of IQ. (B) In the subset of PiB-positive subjects (defined as global mean cortical PiB distribution volume ratio [DVR] ≥ 1.15; n = 55), canonical variate NP performance was inversely related to PiB retention, but no interaction with CR was observed, that is, regression lines are nearly parallel. (C) In the subset of NC subjects (n = 66), the relation of canonical variate NP performance to PiB retention did not reach significance; instead, the significant interaction effect with CR resulted in a systematic splaying of the data according to CR, identified as strata of AMNART IQ. (D) In the further subset of NC subjects with data from the more challenging MCT (n = 34), both a significant main effect of PiB on NP performance and a significant interaction with CR were observed. NC-MCT = normal control Memory Capacity Test.

A post hoc multiple regression of the NP canonical variate on the set of predictors confirmed a significant (*p* < 0.001) partial negative relation of precuneus PiB to the NP variate with a significant, positive coefficient for the interaction term (*p* < 0.03; Table 3), analogous to what was found for the canonical loadings. (Correlations, ie, loadings of each individual predictor variable, ie, age, education, rAMNART, precuneus PiB, and PiB × rAMNART, with their own canonical variates are given in Table 2, along with regression coefficients and significance values relative to their relation to the NP canonical variates.) Plots of the NP variate versus precuneus PiB (Fig 1A, unadjusted for age and education) indicate the main effect as a net downward slope, and an accompanying interaction effect as a splaying of the individual regression lines. (The predicted model, adjusted for age and education, for the full sample is shown in Supplementary Fig S1.) All the above findings were virtually the same if a measure of global amyloid deposition was included as an additional covariate in the predictor set (data not shown), suggesting anatomical specificity of the findings for the precuneus ROI and not phenomena that are spuriously tied to global amyloid.

**Table 2:** Correlations (Loadings) of Predictor Variables with Predictor Canonical Variate, and Regression Analysis of the NP Canonical Variate on Predictors: Full Sample

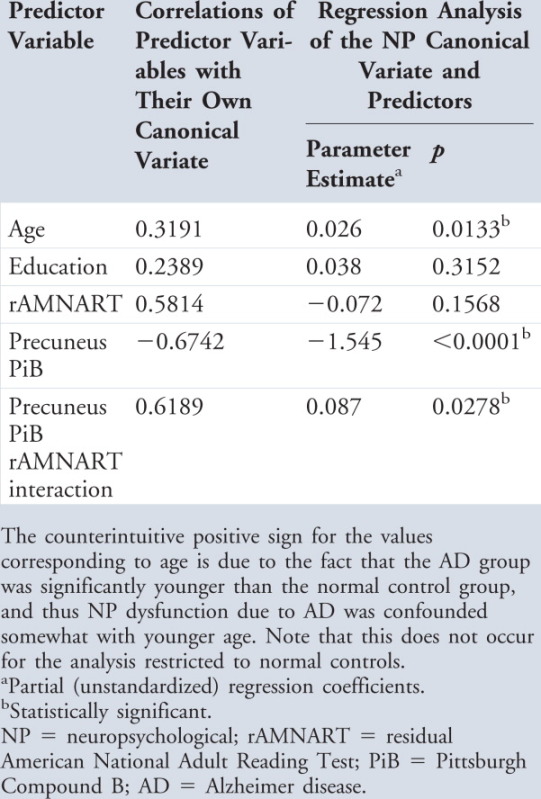

**Table 3:** Multiple Regression Analyses of Individual NP Tests and the NP Canonical Variate on Precuneus PiB Retention and Its Interaction with rAMNART, Covarying Age, Years of Education, and rAMNART

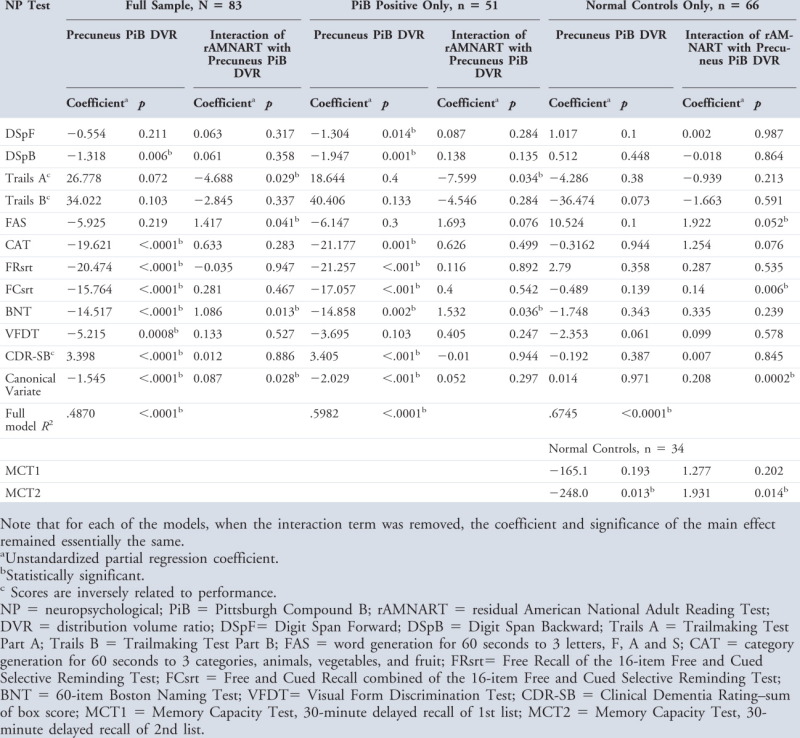

Additional post hoc multiple regressions of individual NP tests on the predictor set revealed significant inverse relationships of precuneus PiB retention with performance on Digit Span Backward, CAT, FRsrt, FCsrt, BNT, VFDT, and CDR-sum of box score. For example, the BNT score was reduced on average by about 14 points for each unit of increase in PiB DVR. Even in the case of the few NP tests showing nonsignificant effects in this regard, the coefficient for precuneus PiB was in the predicted direction (Table 3). In addition, post hoc multiple regressions of individual NP tests on the predictor set revealed significant interactions of PiB retention and rAMNART for Trails A, FAS, and BNT, with coefficient signs in the direction predicted, indicating that high CR suppressed the magnitude of the inverse relation of PiB retention to cognitive test performance. There was no significant interaction of PiB retention and rAMNART on tests of memory, category generation, working memory, visuospatial perception, and CDR status, although virtually all coefficients for the interactions were in the predicted direction. We also evaluated whether the main effect of PiB was observed without the interaction term in the model, and found that precuneus PiB had a highly significant (*p* < 0.0001) inverse relation to the canonical variate, as expected.

### Analyses in Subsample of PiB: Positive Subjects (n = 51)

We performed the same analyses as above on the subset of PiB-positive subjects. Results were virtually identical to those in the full sample (relevant canonical *r* = 0.77, *p* < 0.01; see Fig 1B and Table 4), except that the interaction of precuneus PiB with rAMNART was not statistically significant in the follow-up regression (*p* = 0.30); it was, however, in the hypothesized direction. Note in Figure 1B that the strata of rAMNART form nearly parallel lines, consistent with the lack of an interaction.

**Table 4:** Correlations (Loadings) of Predictor Variables with Predictor Canonical Variate, and Regression Analysis of the NP Canonical Variate on Predictors: PiB-Positive Subjects

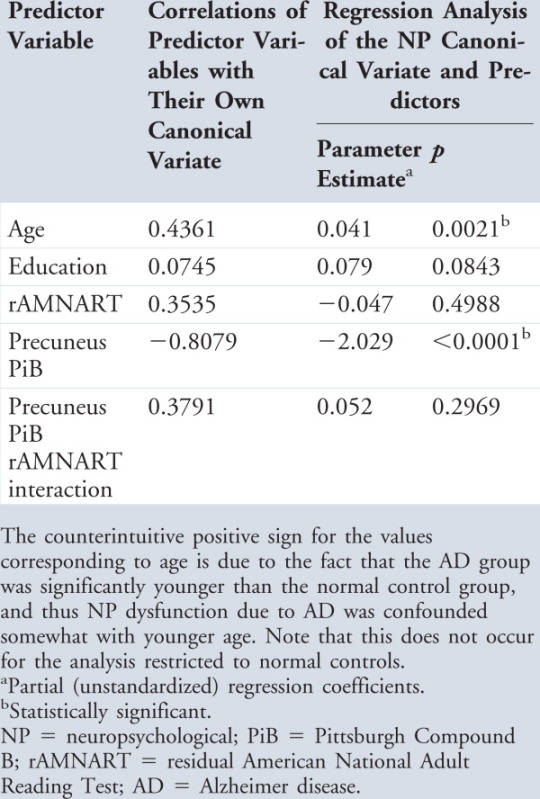

### Analyses in Subsample of Normal Control Subjects (n = 66)

In analyzing the subsample of NCS alone, the largest canonical correlation (*r* = 0.82, *p* < 0.001) was similar to those discussed above in that almost all NP tests loaded at least moderately on it in expected directions (see Supplemental Table S1 and Table 5). For the predictor variable set, the loading for PiB retention (0.10) (see Table 5) suggested that there was little or no overall relationship between amyloid burden and performance on the NP tests. Follow-up multiple regression confirmed no significant, independent relationship of precuneus PiB retention to the NP canonical variate (*p* = 0.97) when the interaction with rAMNART was also included in the model (see Table 3). When the interaction term was not included in the multiple regression model, the main effect of PiB predicting the NP variate was also nonsignificant.

**Table 5:** Correlations (Loadings) of Predictor Variables with Predictor Canonical Variate, and Regression Analysis of the NP Canonical Variate on Predictors: Normal Controls

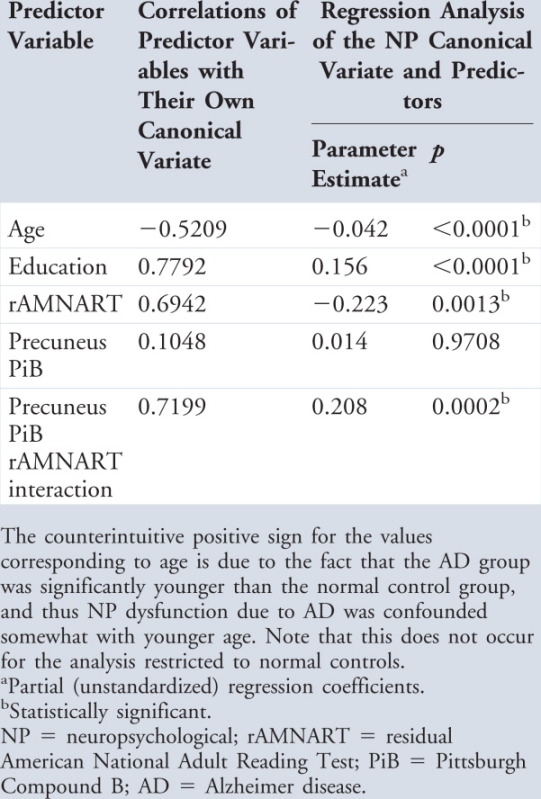

However, the canonical correlation analysis confirmed in the NCs that the interaction of PiB and CR had a large positive loading in the canonical variate (0.72) (see Table 5), and in the follow-up regression, this interaction had a significant (*p* < 0.001), positive coefficient (see Table 3). Thus, the relationship of precuneus amyloid deposition and NP performance was significantly modified by cognitive reserve, as was the case for the larger sample including AD patients. When NP performance (unadjusted for age and education) versus PiB retention is plotted separately for 3 strata of CR, a succession of sloped regression lines can be seen (see Fig 1C), indicating that NP is inversely related at the lowest stratum of CR, but that the relation flattens at higher strata of CR. Thus, in this lower range of PiB retention, at the opposite end of the continuum compared with the PiB-positive subgroup, the overall relation of PiB to NP tests is actually strongest of the 3 groups analyzed (canonical *r* = 0.82, percent variance = 67%; see Table 2). However, here the converse of what was found for the PiB-positive subgroup occurred; it was the interaction effect that dominated and neutralized the net negative main effect of PiB to NP performance, rather than the other way around. Although PiB binding in our normal control sample was skewed toward subjects with lower levels of precuneus PiB binding, it could be argued that the findings were being driven by only a few subjects. However, a large number of cases (30 NCS, 45% of the NCS group) with extensive amyloid deposition suggested that our findings in the normal control sample were not being driven by a small number of cases.

### Analysis of Memory Capacity Test in Normal Control Subjects (n = 34)

The observation that PiB retention is systematically related to cognition in NCs is an important observation and warranted further exploration. Therefore, a more challenging memory test, second list learning during free recall at 30 minutes, was evaluated in a subset of NCs who were available for additional testing. These data came from a behavioral session that was independent from those analyzed above. As hypothesized, we replicated both the significant main effect, that precuneus PiB was inversely related to performance (*p* < 0.01), and the significant interaction with rAMNART (*p* < 0.01) that attenuated the inverse effect at higher CR (see Table 3 and Fig 1D).

### Anatomic Specificity of Amyloid Pathology and Relationship to NP and CR

For both frontal and occipital ROIs, higher PiB retention was associated with poorer performance on the group of 11 NP tests, combined as a canonical variate. For both ROIs, a significant canonical variate indexing good NP performance and loading moderately on most of the NP tests in predicted directions was negatively related to PiB at the ROI. For the frontal ROI, the loading for PiB retention was *r* = −0.61, and for the occipital, *r* = −0.80. Multiple regressions indicated significant, independent, negative relationships for both the frontal (*p* < 0.0001) and occipital (*p* < 0.0001) PiB retention to their respective NP canonical variates. However, for both these ROIs, the interaction of PiB retention with CR was not significant (frontal *p* = 0.82, occipital *p* = 0.79), as it was in the case of the precuneus ROI.

## Discussion

We report that amyloid deposition is associated with reduced cognitive performance among clinically normal individuals (CDR = 0), and that the relationship is systematically weaker in subjects with higher CR. Clinically normal individuals with higher CR have less susceptibility to amyloid-related impairment than those with lower CR. We confirmed the interaction with CR across a range of cognitive performance including patients with mild AD,^35^, ^36^ but found that the subset of subjects above the threshold for amyloid positive seem to derive less benefit from CR, as the main effect of declining performance with increasing amyloid overwhelmed the weaker, moderating effect of CR. Our findings are consistent with epidemiological data suggesting that higher education and occupational attainment is associated with decreased risk for AD,^32^ with postmortem data relating AD pathology to cognitive function during life in clinically normal individuals,^30^ and with CR modification for amyloid plaque-related cognitive function, but not for tangle-related function.^28^

It is not known whether deposited amyloid, soluble forms of β-amyloid, or other associated pathologies are directly responsible for impairment or the effect of CR. However, our findings strengthen existing evidence that brain amyloid burden measured with PET correlates with level of cognitive impairment.^7^, ^16^, ^20^, ^64^ Similarly, although high CR permits individuals to tolerate encroaching pathology, it is not known whether this comes about because of higher synaptic or neural capacity or greater efficiency in cognitive strategies or network engagement that may have neuroprotective effects related to CR. Conversely, those with lower CR may have been exposed to developmental circumstances that prevent the achievement of higher CR, thus producing increased vulnerability to amyloid's neurotoxic effects. It is also possible but unknown whether the transition from soluble to deposited amyloid itself provides a form of reserve by sequestering putatively more toxic forms of β-amyloid.^65^, ^66^ What does seem clear, however, is that attempts to relate amyloid PET to NP performance or to treatment-related changes in NP performance should be interpreted against the background of each subject's level of CR.

The relationship of NP performance to amyloid burden was much less obvious in subjects with high levels of CR when the NP canonical variate was used. Because we suspected that ceiling effects could obscure the relationship, we considered the possibility that more challenging test instruments could improve our ability to detect interaction effects at higher levels of CR. We found in an independent test session that the use of the MCT, an episodic memory test with no evidence of ceiling effects in our sample, permitted us to discover not only the interaction of amyloid with CR, but also the main effect of reduced performance with higher levels of amyloid.

High amyloid burden in the precuneus was related in the full sample of subjects to poorer performance across multiple domains of NP function, including tests of working memory (Digit Span Backward), episodic memory (FRsrt and FCsrt), semantic processing (CAT), naming (BNT), and visuospatial perception (VFDT). In contrast, tests that measured speed of processing (eg, Trails A) and letter fluency (ie, FAS) were not associated with PiB retention in the precuneus but, similar to the findings of Bennett et al in analyses of postmortem data,^29^ did have a significant interaction with CR (see Table 3). CR could have a stronger effect on particular cognitive tests but not others, because some tests may permit individuals to use a broader range of alternate cognitive strategies that are more accessible to those with higher CR.^67^, ^68^ For example, some tasks, such as Trails A and FAS, require more primitive sequencing and phonemic search strategies that may still be accessible to those with higher CR, allowing for the successful completion of the task even in the context of extensive amyloid deposition. The clinical literature has widely reported that letter fluency tasks, such as FAS, are much better preserved in patients with AD.^45^, ^69^, ^70^

Our examination of specific ROIs indicated that PiB retention in the frontal region, which has been reported to bear a substantial amyloid burden,^7^, ^10^, ^13^, ^19^ was related to NP performance, but there was no interaction with CR. This finding suggests that, compared with precuneus, frontal amyloid deposition may be linked to impairment and disease severity, but the protective effect of CR is not as readily observable. This finding differs from Kemppainen et al,^37^ who found that highly educated AD patients showed significant increases in PiB uptake in the frontal region compared with less educated AD patients, perhaps because their sample included only impaired subjects. Our finding that CR did not attenuate amyloid-related performance in frontal regions may relate to reports that frontal PiB retention was not as strongly related to atrophy or gray matter loss^20^, ^58^ or to fluorodeoxyglucose hypometabolism.^7^, ^8^, ^15^

Lower strata of CR were underrepresented in our sample of NCS (mean IQ = 121.9), and all NCS in the lowest IQ quartile had lower levels of PiB retention (see Fig 1C). One possible explanation for this is consistent with our hypothesis that individuals in the lowest stratum of CR would have greater levels of impairment as PiB retention increases than those in higher strata. Therefore, lower CR subjects with higher levels of amyloid would be less likely to be classified as clinically normal. To evaluate this possibility, we are currently recruiting subjects specifically targeting the lower strata of CR. If our observations are extrapolated to these individuals, however, they would be the most clinically impaired and least able to participate.

Although we chose to use the AMNART IQ as a proxy of reserve, we realize it has limitations. For example, AMNART IQ measures only 1 aspect of CR, namely verbal ability level, but there are other factors that may contribute to CR, such as early educational experiences, late-life cognitive activities, life-style factors, occupation, and socioeconomic status.^28^, ^71^ We also recognize that the AMNART IQ may not be an accurate measure of premorbid ability for all individuals, particularly those with reading difficulties or non–English-speaking participants. Although all subjects in our sample were English speaking, and no one had a history of learning disabilities, this limits the use of AMNART IQ for all populations. Finally, education has been used successfully in several other PiB studies as a proxy of reserve,^22^, ^36^ but we found that in our older population, a bias occurred where women did not have the same educational advantages as men. In the past, we found a similar bias directly comparing education and AMNART IQ in older individuals.^72^ When education was used as an interaction variable with precuneus PiB, we also found a significant main and interaction effect with CR, as others have reported.^36^, ^37^ However, when the interaction of rAMNART IQ with CR was added to the model, the interaction with education became nonsignificant. Possibly, AMNART IQ may be closer to the underlying operative of the CR concept, and education may be sharing variance as a confounded correlate. In the future, it will be important to explore further the many dimensions of CR to more closely approximate this concept.

Another limitation of this report is the lack of apolipoprotein E (APOE) genotyping in all subjects. Reiman et al^73^ have recently reported that fibrillar beta-amyloid is significantly associated with APOE epsilon 4 carrier status in cognitively normal older individuals. It is possible that our findings in the normal control sample may have been attributable to APOE variants. In the entire sample of 83 subjects, 43 had APOE genotyping. We reran the above canonical analysis and included APOE genotyping with 1 or no copies of the APOE epsilon 4 allele as a dummy-coded predictor variable. We found that APOE status was not a significant predictor of the NP canonical variate, whether it was included as an additional predictor in the canonical analysis or merely introduced as an additional covariate in the follow-up regressions. Among the subjects with genotyping, we found a trend (*p* = 0.10) in precuneus PiB deposition, with E4 carriers having slightly more amyloid (DVR = 1.35 ± 0.3) than non-E4 carriers (DVR = 1.21 ± 0.2). However, the lack of a significant finding is most likely related to small sample size. Future work will be required to explore the association of genetic factors including APOE genotype to determine if APOE makes an independent contribution to our observed findings of CR modification.
