# Supplementary material for: Cognition, Reserve, and Amyloid Deposition in Normal Aging
Source: Ann Neurol. 2009 Oct 27;67(3):353–64. doi: 10.1002/ana.21904 (PMC3074985; doi:10.1002/ana.21904)
Supplement: Supplementary file 1 [file ana0067-0353-SD1.doc]

Supplemental Table S1. The canonical correlation loadings of the individual NP variables with the NP canonical variates calculated in each subsample.

|  | Full Sample, N=83 | | PiB-Positive, N=55 | | Normal Control, N=66 | |
| --- | --- | --- | --- | --- | --- | --- |
| NP Tests | CanVar1_NP* | CanVar2_NP* ^#^ | CanVar1_NP | CanVar2_NP ^#^ | CanVar1_NP ^#^ | CanVar2_NP |
|  |  |  |  |  |  |  |
| DSp F | 0.23 | **0.56** | 0.18 | **0.66** | **0.53** | 0.38 |
| DSp B | -0.05 | **0.65** | -0.02 | **0.77** | **0.36** | 0.20 |
| Trails A | 0.03 | **-0.43** | -0.14 | **-0.40** | **-0.49** | 0.60 |
| Trails B | 0.01 | **-0.39** | -0.06 | **-0.46** | **-0.41** | 0.12 |
| FAS | 0.35 | **0.69** | 0.49 | **0.56** | **0.61** | 0.62 |
| CAT | -0.10 | **0.72** | 0.05 | **0.67** | **0.72** | 0.13 |
| FRsrt | -0.51 | **0.58** | -0.32 | **0.62** | **0.36** | -0.17 |
| FCsrt | -0.52 | **0.67** | -0.29 | **0.71** | **0.50** | -0.21 |
| BNT | -0.06 | **0.84** | 0.13 | **0.71** | **0.75** | 0.31 |
| VFDT | -0.27 | **0.48** | -0.09 | **0.38** | **0.29** | -0.22 |
| CDR-SB | 0.58 | **-0.62** | 0.36 | **-0.63** | **-0.10** | 0.14 |
|  |  |  |  |  |  |  |
| Predictors | *CanVar1_PV** | *CanVar2_PV*^#^* | *CanVar1_PV* | *CanVar2_PV^#^* | *CanVar1_PV^#^* | *CanVar2_PV* |
| Age | -0.16 | **0.32** | -0.01 | **0.44** | **-0.52** | 0.73 |
| Education | 0.53 | **0.24** | 0.42 | **0.07** | **0.78** | 0.15 |
| rAMNART | 0.63 | **0.58** | 0.81 | **0.35** | **0.69** | 0.61 |
| Precuneus PIB | 0.69 | **-0.67** | 0.48 | **-0.81** | **0.11** | 0.21 |
| Precuneus X rAMNART | 0.68 | **0.62** | 0.86 | **0.38** | **0.72** | 0.60 |

^#^Refers to the canonical correlation that was used in the analysis.

CanVar1_NP refers to canonical variate for the NP tests, corresponding to the first canonical correlation. Similarly CanVar2_NP for the canonical variate for the NP tests associated with the second canonical correlation.

Two statistically significant canonical correlations emerged from the data that were of about equal strength (p< 0.0001 and p<0.0003). Each of these consists of a pair of variates, which were the neuropsychological variate, CanVar_NP, and the predictor variate, CanVar_PV. Thus for each of the analyses (Full sample, PiB-positive, and Normal Control) 2 pairs of canonical variates are shown. Note that the two pairs of canonical variates are forced by derivation to be uncorrelated and unconfounded with each other, and therefore what one shows has no bearing on the validity and meaning of the other. For each of the analyses (Full sample, PiB-positive, and Normal Control), we elected to use one of the two pairs of canonical variates (shown in boldface type), and our choice was based on the following:

1) Only one of the two reflected a global cognitive dysfunction that correlated with virtually all the NP measures we employed (i.e, the high correlation loadings in bold-face type in Table S1), whereas the other (not in boldface) correlated at levels generally much lower, with high loadings on only a few of the NP tests (FRsrt, FCsrt, and CDR-SB).

2) Only one of the two pairs of canonical variates was also correlated consistently with the predictor variables, such that amyloid in the precuneus was negatively related to cognitive performance, and that there was also a modifying effect of cognitive reserve, such that for increasingly higher strata of cognitive reserve, the negative relation of amyloid to cognitive function became weaker and weaker. In contrast, the other pair of variates (not in boldface) indicated that although increased amyloid predicted worse performance on (a few of) these measures, there was a counterintuitive relation to cognitive reserve such that the deleterious effect of amyloid on performance on these few measures appeared to get even worse at higher cognitive reserve.


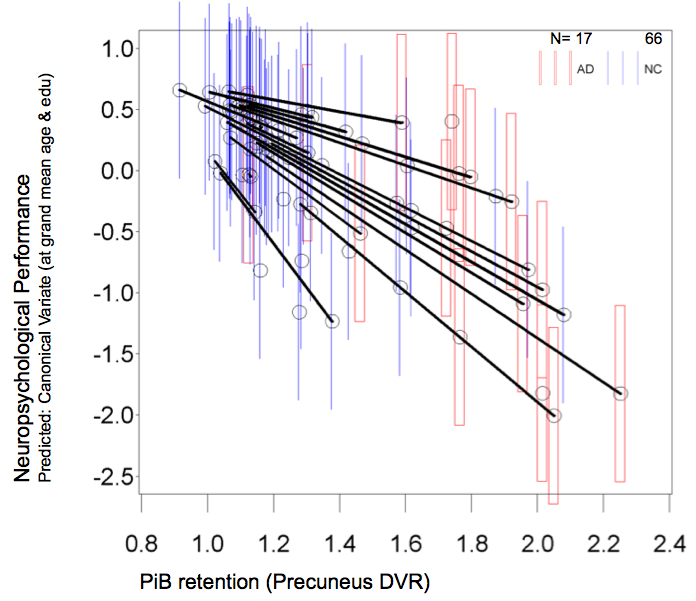
Figure S1

**Figure 1S. Regression model-predicted neuropsychological performance vs. precuneus PiB retention plotted at strata of cognitive reserve (rAMNART IQ).** Circles are predicted values of canonical variate of neuropsychological (NP) tests from regression on precuneus PiB, rAMNART IQ, age, education and the interaction of precuneus PiB and rAMNART for the full sample (N=83; R-squared=0.49, root mean square error=0.74). Diagonal lines connect points with equal rAMNART IQ values (unit rounded) where higher lines with more positive/less negative slopes represent progressively higher rAMNART values (-35 to 13). (A circle without a line represents a person whose rAMNART value did not match any other person’s). For this illustration, age and years of education are set at constants equal to their grand means (72 and 16 years, respectively). The vertical error bars have lengths equal to the root mean square error from the model, above and below each point. The thin vertical error bars indicate normal control subjects and the wider, open bars indicate AD patients. High CR suppresses the otherwise negative effect of PiB (interaction p< 0.03).
